# Complete Genome Sequence of Rahnella aquatilis MEM40, a Plant Growth-Promoting Rhizobacterium Isolated from Rice Rhizosphere Soil, with Antagonism against Magnaporthe oryzae and Fusarium graminearum

**DOI:** 10.1128/MRA.00651-20

**Published:** 2020-09-10

**Authors:** Xiaowen Sun, Wei Ma, Yin Xu, Xinmeng Jin, Hong Ni

**Affiliations:** a State Key Laboratory of Biocatalysis and Enzyme Engineering, Hubei Collaborative Innovation Center for Green Transformation of Bio-resources, School of Life Sciences, Hubei University, Wuhan, People’s Republic of China; b State Key Laboratory of Agricultural Microbiology, Huazhong Agricultural University, Wuhan, People’s Republic of China; University of California, Riverside

## Abstract

Rahnella aquatilis strain MEM40 is a plant growth-promoting rhizobacterium (PGPR) with antagonism against Magnaporthe oryzae and Fusarium graminearum that was isolated from rice rhizosphere soil in Hubei, China. Here, we report its complete genome sequence, which will increase our understanding of the mechanisms of plant growth promotion and biocontrol.

## ANNOUNCEMENT

**FIG 1:**
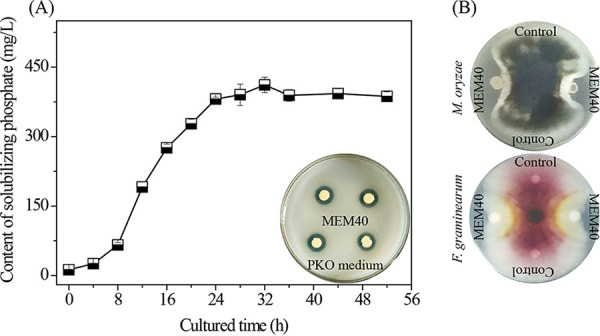
Phosphate-solubilizing and antifungal capacities of MEM40. (A) Phosphate-solubilizing capacity of MEM40 in Pikovskaya liquid medium [10.0 g glucose, 5.0 g Ca_3_(PO_4_)_2_, 0.5 g (NH_4_)_2_SO_4_, 0.2 g NaCl, 0.1 g MgSO_4_·7H_2_O, 0.2 g KCl, 0.5 g yeast extract, 0.002 g MnSO_4_·H_2_O, and 0.002 g FeSO_4_·7H_2_O, pH 7.0 to 7.4] (the maximum phosphate-solubilizing capacity reached 411.56 mg/liter). The inset shows a transparent zone of MEM40 on Pikovskaya solid medium. (B) Antifungal test of MEM40 against *M. oryzae*, the causal agent of rice blast disease (13), and F. graminearum, the causal agent of wheat *Fusarium* head blight (14), on peptone-dextrose agar (PDA) medium (200.0 g potato, 15.0 g glucose, and 15.0 g agar, pH 7.0 to 7.4).

Rahnella aquatilis is a Gram-negative facultative anaerobic nonpigmented rod-shaped bacterium belonging to the *Enterobacteriaceae* that has been found in a variety of environments (1, 2). This species can act as a plant growth-promoting rhizobacterium (PGPR) to provide nutrients for plant development (3, 4) and can also be used as a biological control agent against some plant diseases, including grapevine crown gall (5), apple fire blight (6), and fruit storage rots (7). Strain MEM40 was isolated from rice-wheat rotation soil (Hubei, China) using the 10-times dilution coating method on Pikovskaya solid medium. The single colony with a transparent zone was grown in Luria-Bertani medium overnight at 28°C. PCR was performed using the universal primers 27F (5′-AGAGTTTGATCCTGGCTCAG-3′) and 1492R (5′-TACGGCTACCTTGTACGACTT-3′). Phylogenetic analysis of the 16S rRNA gene identified MEM40 as an *R. aquatilis* strain. MEM40 possessed phosphate-solubilizing capacity (Fig. 1A) and antagonistic effects against *Magnaporthe oryzae* and Fusarium graminearum (Fig. 1B). The complete genome sequence of MEM40 may be used in comparative genomics, functional gene searches, and engineering bacteria in the future.

A single colony was grown in Luria-Bertani medium overnight at 28°C. Genomic DNA was extracted using a bacterial genomic DNA extraction kit (Solarbio, Beijing, China) and sheared into ∼10-kb fragments with a gTUBE device (Covaris, USA). After single-strand overhang removal, DNA damage repair, end repair, and 3′ adenylation, the fragments were linked with single-molecule real-time (SMRT) adaptors using the SMRTbell Express template preparation kit v2.0 (Pacific Biosciences, USA) and purified with AMPure PB beads. DNA concentration and library fragment size were measured with Qubit v3.0 and the Agilent 2100 bioanalyzer system. The library was sequenced with the Sequel sequencing kit v2.1, and sequencing data were processed using SMRT Link v5.0 (8, 9). The read library contained 2,306,916,870 bp with an average subread length of 8,183 bp, a subread *N*_50_ value of 10,647 bp, and an average coverage of 459×. HGAP4 software (HGAP genome length, 5,500,000 bp; HGAP seed coverage, 30; HGAP seed length cutoff, –1; minimum confidence, 40; minimum coverage, 5) (10) and Canu v1.6 (genome size, 5.5 Mbp; corrected error rate, 0.02) were used for assembly and self-correction. Minimus2 software (with the parameters overlap, 40 bp; conserr, 0.06; minid, 94; and maxtrim, 20 bp) was used for cyclization. The MEM40 genome consisted of a circular chromosome and two circular plasmids, and the genome size was 5,596,168 bp with a subread *N*_50_ value of 5,596,168 and G+C content of 52.2%. The publicly available sequence was annotated using NCBI PGAP. The clustered regularly interspaced short palindromic repeat (CRISPR) structures and genomic islands (GIs) were predicted using CRT (11) and IslandPath (12) software, respectively.

The circular chromosome consisted of 5,021,016 bp (G+C content, 52.3%) containing 4,467 protein-coding genes, 45 pseudogenes, 22 rRNA genes, 77 tRNA genes, 6 CRISPRs, and 11 GIs (Table 1). The circular megaplasmid pMEM40-1 consisted of 525,842 bp (G+C content, 52.1%) containing 470 protein-coding genes, 8 pseudogenes, and 1 CRISPR (Table 1). The circular plasmid pMEM40-2 consisted of 49,310 bp (G+C content, 37.4%) containing 27 protein-coding genes and 3 pseudogenes.

**TABLE 1:** CRISPR and GI positions of the MEM40 genome

Item	Nucleotide locations of chromosomes	Nucleotide location of plasmid:	
		pMEM40-1	pMEM40-2
CRISPRs	10,161–10,313; 605,085–605,242; 1,545,386–1,545,528; 4,610,085–4,612,034; 4,613,182–4,613,989; 4,622,496–4,622,644	3,673–4,459	None
GIs	435,106–444,072; 630,109–651,491; 1,450,010–1,493,385; 1,967,133–1,987,278; 2,131,920–2,198,015; 2,755,996–2,770,900; 3,341,310–3,347,746; 3,963,003–3,979,126; 4,712,350–4,737,074; 4,782,356–4,818,109; 4,929,997–4,936,332	None	None

### Data availability.

The genome assembly has been deposited in GenBank under the accession number GCA_004328445.1. The complete genome sequence of MEM40 has been deposited in GenBank under the main chromosome accession number CP036490.1, and the sequences of the plasmids pMEM40-1 and pMEM40-2 have been deposited under accession numbers CP036488.1 and CP036489.1, respectively. Sequence data have been deposited in the Sequence Read Archive under the accession number SRP267417.
